# Effects of a *Rosmarinus officinalis* L. Extract and Rosmarinic Acid in Improving Streptozotocin-Induced Aortic Tissue Damages in Rats

**DOI:** 10.3390/nu17010158

**Published:** 2024-12-31

**Authors:** Irina Ielciu, Gabriela Adriana Filip, Alexandra C. Sevastre-Berghian, Ioana Bâldea, Neli-Kinga Olah, Ramona Flavia Burtescu, Vlad Alexandru Toma, Remus Moldovan, Ilioara Oniga, Daniela Hanganu

**Affiliations:** 1 Department of Pharmaceutical Botany, Faculty of Pharmacy, “Iuliu Haţieganu” University of Medicine and Pharmacy, 400337 Cluj-Napoca, Romania; irina.ielciu@umfcluj.ro; 2 Department of Physiology, Faculty of Medicine, “Iuliu Haţieganu” University of Medicine and Pharmacy, 400006 Cluj-Napoca, Romania; berghian.alexandra@umfcluj.ro (A.C.S.-B.); ioana.baldea@umfcluj.ro (I.B.); remus_ri@yahoo.com (R.M.); 3 Department of Pharmaceutical Chemistry, Faculty of Pharmacy, “Vasile Goldiş” Western University of Arad, 310414 Arad, Romania; olah.neli@uvvg.ro; 4 PlantExtrakt Ltd., Rădaia, 407059 Cluj-Napoca, Romania; ramona.burtescu@plantextrakt.ro; 5 Department of Molecular Biology and Biotechnology, Babes-Bolyai University, 400371 Cluj-Napoca, Romania; vlad.toma@ubbcluj.ro; 6 Department of Pharmacognosy, Faculty of Pharmacy, “Iuliu Haţieganu” University of Medicine and Pharmacy, 400010 Cluj-Napoca, Romania; ioniga@umfcluj.ro (I.O.); dhanganu@umfcluj.ro (D.H.)

**Keywords:** *Rosmarinus officinalis* L., rosmarinic acid, diabetes mellitus, streptozotocin-induced cardiac damage

## Abstract

Background/Aim: *Rosmarinus officinalis* L. (*R. officinalis*) is an aromatic medicinal species with important nutraceutical potential, having rosmarinic acid (RA) as one of its main metabolites. The present study aims to evaluate the effects of an extract obtained from the leaves of this species and of its main metabolite in improving the streptozotocin-induced damage of hearts and aorta of diabetic rats. Methods: The leaves of the species were used to obtain a hydroethanolic extract, which was analyzed using the LC/MS method. Diabetes mellitus was induced by intraperitoneal streptozotocin administration in rats. After two weeks, oxidative stress parameters were evaluated from the heart and aorta homogenates. NOS3, AMPK, and adiponectin levels were quantified using ELISA tests, and thoracic aorta rings were isolated for contractility evaluation in the organ bath. Phospho-NF-κB, NRF2, HIF1 alfa, iNOS, and glyceraldehyde 3-phosphate dehydrogenase (GAPDH) quantification were performed using the Western blot technique. Results: Carnosic acid, together with rosmarinic acid, were proven to be the main metabolites identified in the composition of the tested extract. Administration of the extract and of RA improved the relaxation response to acetylcholine and the redox status, with the reduction in malondialdehyde (MDA), nitric oxide synthase 3 (NOS 3), AMP-activated protein kinase (AMPK), adiponectin, reduced (GSH) and oxidized glutathione (GSSG) levels, and superoxide dismutase (SOD) activity. RA significantly enhanced the expression of HIF 1α, NRF2, and pNFkB in the heart. Conclusions: Administration of the *R. officinalis* extract and of RA-alleviated oxidative stress, proving vascular and cardiac antioxidant properties in the hearts and aorta of diabetic rats.

## 1. Introduction

Rosmarinic acid (RA) is known as one of the most valuable secondary metabolites, being largely distributed in numerous vegetal sources, mostly included in the Lamiaceae family [1], where it was reported even before having its structure elucidated [2]. It is a molecule with important applications, ranging from cosmetics and food preservatives to pharmaceuticals [1,3]. Its antidiabetic, anti-inflammatory, hepatoprotective, anticancer, cardioprotective, and antimicrobial activities have been broadly investigated but continue to present interest to researchers, especially in the pharmaceutical domain [1,2,3,4,5]. The species that have this valuable compound in significant amounts in their composition include *Thymus* sp., *Salvia* sp., *Lavandula* sp., and *Rosmarinus officinalis* [1,2].

*Rosmarinus officinalis* L. (syn. *Salvia rosmarinus* Spenn.—rosemary) is a species that originated in the Mediterranean basin; however, due to its high adaptability, it has become easily cultivated throughout the whole world [6,7]. It is a perennial shrub with evergreen, needle-like leaves and white, pink, purple, or blue small flowers that can be used fresh, dried, as an extract or as an essential oil [8,9,10,11]. The vegetal medicinal product is represented by its leaves, which give the species its specific aroma. It is used as a flavoring agent, spice, and preservative [6] and is also largely used as a therapeutic agent, with important pharmacological activities, such as antioxidant, antimicrobial, antidiabetic, anti-inflammatory, and antiproliferative ones [12,13,14,15]. All these activities are due to its numerous bioactive compounds, such as flavonoids, di- and triterpenoids, monoterpenes, sesquiterpenes, hydroxycinnamic derivatives, and other minor compounds [6,8,9]. The composition of its essential oil may vary depending on the pedoclimatic conditions and the age of the plant and extraction and preparation conditions of its different products (e.g., storage, drying, solvent type and concentration, temperature) may also influence its phytochemical profile [6,8,16,17]. The volatile compounds in the composition of its essential oil are represented by ketone monoterpenes and sesquiterpenes such as camphor, borneol, linalool, or humulene [15]. The nonvolatile fraction consists of phenolic acids, flavonoids and phenolic terpenes, organic acids, diterpenoids, and triterpenoids. Among phenolic acids, caffeic, p-coumaric, ferulic, gallic, quinic, and syringic acids are found in important amounts, while phenolic triterpenes are represented by betulinic, oleanolic, and ursolic acids are found in the composition of *R. officinalis* [3,16,17]. All these have helped extracts of this species and its essential oils to receive recognition, attesting to their applicability and safety for the food and medicinal industry [7].

Diabetes mellitus (DM) is a complex metabolic pathology characterized by hyperglycemia fluctuations due to the malfunctioning of insulin production, its activity, or both [18]. It is one of the major burdens for health systems worldwide, a fact that is evidenced by its continuously rising prevalence in adult populations, as estimated by the World Health Organisation (WHO) [19]. DM is widely agreed as constituting a major risk for cardiovascular diseases that have mechanisms still to be investigated. One of the most frequent is related to the important production of reactive oxygen species (ROS) due to hyperglycemia, radicals involved in endothelial dysfunction, and vascular damage [19,20]. In this context, antioxidants, especially the ones in nutraceuticals, play an important role and, as in the last years, the need to offer treatments from natural sources has significantly increased [19,21]. Numerous nutraceuticals have proven highly effective in the management of DM and its complications, exhibiting anti-radical and anti-inflammatory effects due to the ability of key enzymes such as alpha-glucosidase and lipase that can improve pancreatic function and insulin release. Among these nutraceuticals, one of the most promising appears to be the *R. officinalis* [22].

Having taken all this into consideration, the present study aims to offer further scientific arguments sustaining the biological potential of RA and of an *R. officinalis* hydroethanolic extract (Ro) in alleviating the streptozotocin-induced cardiac and vascular tissue damage. Therefore, the main goal of the present research was to study the in vivo protective biological activity of *R. officinalis* and RA on the heart and aorta against oxidative stress and enzyme activity in rats with DM. The effects of the *R. officinalis* extract and of RA on the aortic rings on contractile responses to phenylephrine and acetylcholine were analyzed. Additionally, the malondialdehyde (MDA), nitric oxide synthase 3 (NOS 3), AMP-activated protein kinase (AMPK), adiponectin, reduced (GSH) and oxidized glutathione (GSSG) levels, and superoxide dismutase (SOD) activity in the hearts and aortas of diabetic rats were assessed. The studies were carried out on rats with DM experimentally induced by the administration of streptozotocin, treated with an extract obtained from *R. officinalis*, as well as RA, in order to comparatively assess their biological effects. The novelty of the present study is represented by the fact that it is, to the best of our knowledge, the only study to compare the effects of both the *R. officinalis* extract and of RA, assessing their effects on the cardiac tissue damage induced in DM and its complications, offering evidence that may justify their future potential use in the treatment of these pathologies.

## 2. Materials and Methods

### 2.1. Chemicals and Reagents

O-phthalaldehyde, 2-thiobarbituric acid, and Bradford reagent were purchased from Merck KGaA (Darmstadt, Germany). ELISA tests for NOS3, AMPK, and adiponectin were purchased from Elabscience (Houston, TX, USA). Antibodies against transcription factors phospho-NF-κB, nuclear factor erythroid 2–related factor 2 (NRF2), hypoxia-inducible factor-1 (HIF1) alfa, inducible nitric oxide synthase (iNOS) enzymes and gyceraldehyde 3-phosphate dehydrogenase (GAPDH) were obtained from Santa Cruz Biotechnology (Delaware Ave, Santa Cruz, CA, USA). Glucose levels were monitored by using a kit provided by Diagnosticum Rt (Budapest, Hungary). All solvents and reagents used for analysis were of analytic-grade purity and were obtained from Merck KgaA (Darmstadt, Germany). Rosmarinic acid and other references used in the LS-MS method were also of analytic-grade purity and were obtained from Phytolab (Vestenbergsgreuth, Germany).

### 2.2. Vegetal Material

The leaves of R. officinalis were collected from cultures in Oradea, Romania (Bihor county). The plant material was identified by Lecturer Irina Ielciu at the Department of Pharmaceutical Botany of the Faculty of Pharmacy of the “Iuliu Haţieganu” University of Medicine and Pharmacy Cluj-Napoca. The vegetal material was air dried at room temperature, labeled, and preserved appropriately to be used in the experiments provided in the following steps [23,24,25].

### 2.3. Preparation of the Extract

The dry vegetal material (100 g) was ground, and the obtained powder was subsequently subjected to maceration at ambient temperature, according to a method described by the European Pharmacopoeia, using 1000 mL ethanol 70% *V*/*V* as the extraction solvent [26]. The ratio of vegetal product: extraction solvent was 1:10. The obtained mixture was periodically shaken, 20 min per day, for 10 days. The extract thus obtained was used for the identification and quantification of polyphenolic compounds by LC–MS [24,27]. The aqueous extract, obtained through the evaporation of ethanol from the macerate using a rotary evaporator, was used for the in vivo studies [24]. The yield of extraction was 1.99%.

### 2.4. LC–MS Method and Apparatus

A Shimadzu Nexera I LC–MS-8045 (Kyoto, Japan) UHPLC system, equipped with a quaternary pump, an autosampler, an ESI probe, and a quadrupole rod mass spectrometer was used for the LC–MS analysis. Separation was carried out on a Luna C18 reversed-phase column (150 mm × 4.6 mm × 3 µm, 100 Å, Phenomenex-Torrance, California, CA, USA). The temperature of the column was set at 40 °C. The mobile phase was represented by a gradient composed of LC–MS analytical-grade methanol and ultrapurified water, prepared using the Simplicity ultrapure water purification system (Merck Millipore, Billerica, MA, USA). The composition of the gradient is described in Table 1. LC–MS analytical-grade formic acid was used as the organic modifier for the mobile phase. The flow rate was maintained at 0.5 mL/min during the analysis, and the total analysis time was 35 min.

Detection was performed on a quadrupole rod mass spectrometer using electrospray ionization (ESI) in negative and positive multiple reaction monitoring (MRM) ion modes. The temperature of the system was set at 300 °C. For vaporization, nitrogen (30 psi and a flow rate of 10 L/min) was used as a drying gas. The potential of the capillary was set at +3000 V and injection volumes were 1 µL for references and 10 µL for samples. Identification of metabolites was performed by comparison of retention times, MS spectra, and transitions between separated metabolites from the extract and references. Identification and quantification were performed based on the main transition from the MS spectra of each individual metabolite. Calibration curves (R^2^ = 0.9964–0.9999) were plotted for the quantification of each metabolite and reference. The method was validated by assessing linearity, precision, and accuracy, in accordance with the International Conference on Harmonization guidelines (ICH). The limit of detection (LOD) and quantification (LOQ) were calculated after the injection of different concentrations of each analyzed metabolite. The accuracy of the method was determined in duplicate using a recovery experiment. All samples were injected in triplicate [28,29].

### 2.5. In Vivo Studies

#### Experimental Design

To study the biological effects of the *R. officinalis* species extract and of pure RA in DM, Wistar rats with experimentally induced DM were used. Female Wistar rats (n = 45) were provided by the Biobase of the “Iuliu Haţieganu” University of Medicine and Pharmacy, Romania. Before the experiment, rats were acclimatized using the following conditions: temperature of 21 °C, light/dark cycle of 12 h/12 h, humidity of 65%, and unlimited access to water. The used feed was the standard one (VRF1). Rats were deprived of food and had access to water 16 h before and 24 h after gavage and the intravenous administration of the extract or of the vehicle. Animals were separated into 4 groups of 9 animals/group. In the last 3 groups, DM was induced through the administration of streptozotocin (STZ) in a single dose of 40 mg/kg body. Various doses of STZ can be used in order to experimentally induce DM, such as a single moderate dose, a single large dose, or multiple low doses [30,31,32,33]. However, it has been reported that a 40 mg/kg dose of IP STZ is optimal for creating diabetes with moderate hyperglycemia in Wistar (male and female) rats. Additionally, our previous experience confirmed that the 40 mg/kg b.w. dose of STZ is appropriate to induce hyperglycemia.

The control group, group 1, represents the animals without DM who received distilled water (C group). The animals with DM were treated with different compounds as follows: group 2—treated with the same amount of distilled water (DM); group 3—treated with 200 mg/kg b.w. R. officinalis extract in distilled water (DM + Ro); and group 4—treated with 20 mg/kg b.w. RA in distilled water (DM + RA). In the first 72 h after STZ administration, blood glucose levels were monitored and animals with glycemia values above 250 mg% were included in the study. The tested compound and the extract were administered daily for 14 days. On day 15, under anesthesia with 90 mg/kg body ketamine and 10 mg/kg body xylazine, the blood, aorta, and heart fragments were collected for biochemical and histopathological analysis. Descendent aorta rings approximately 5 mm in length were also collected and placed in a Krebs–Henseleit solution (KHS) at 4° Celsius. Afterwards, rings were mounted in a tissue bath system (BIOPAC MP150, BIOPAC Systems Inc., Goleta, CA, USA) in a 20 mL KHS solution, heated to 37° Celsius, with the following composition: D-Glucose 2.0 g/L, Magnesium Sulfate 0.14 g/L, Potassium Phosphate 0.16 g/L, Potassium Chloride 0.35 g/L, Sodium Chloride 6.9 g/L, Calcium Chloride 0.28 g/L, Sodium Bicarbonate 2.09 g/L. The isometric force was measured using force transducers connected to the BIOPAC MP150 system and AcqKnowledge software, version 3.9.0 [24,34,35].

### 2.6. Oxidative Stress Evaluation

Oxidative stress parameters were assessed from the heart and aorta tissue homogenates. Oxidative stress was quantified by using the fluorimetric method with 2-thiobarbituric acid and was expressed as nM/mL protein. MDA and SOD activity was assessed using the cytochrome c reduction method while GSH and GSSG levels were measured fluorimetrically using o-phthalaldehyde [24,27,36].

### 2.7. Enzymes and Transcription Factors Assessment

NOS3, AMPK, and adiponectin levels were quantified according to the manufacturer’s instructions using ELISA tests, and the results were expressed as ng/mg protein. Phospho-NF-κB, NRF2, HIF1 alfa, iNOS, and GAPDH evaluation were performed using the Western blot technique. Lysates (20 μg protein/lane) were separated via electrophoresis on 8% SDS PAGE gels under reducing conditions and then transferred to polyvinylidenedifluoride membranes (BioRad) using the Biorad Miniprotean system (BioRad). Blots were then blocked and incubated with antibodies against NOS2 (iNOS), phospho-NF-κB (Ser536) (93H1), NRF2, HIF1 alfa, and GAPDH (Santa Cruz Biotechnology, Heidelberg, Germany) diluted in 1:500. After washing, the blots were incubated with corresponding secondary HRP-linked antibodies (1:1500) (Santa Cruz Biotechnology). Proteins were detected and visualized using Supersignal West Femto Chemiluminiscent substrate (Thermo Fisher Scientific, Rockford, IL, USA) and a Gel Doc Imaging system equipped with an XRS camera and Quantity One analysis software, version 3.0.1. (Biorad). GAPDH was used as a protein loading control [27].

### 2.8. Histological Analysis

The heart and aorta of rats were collected for histological investigation. The tissue samples were fixed in 10% neutral buffered formalin, embedded in paraffin in order to crop 5 μm thick sections. For microscopy examination, sections were stained with hematoxylin–eosin (HE) and analyzed with an Optika B-383LD2 microscope [24].

### 2.9. Statistical Analysis

Data were statistically processed using the GraphPad Prism software, version 6.0 (GraphPad, San Diego, CA, USA). Results were expressed as mean ± standard deviation (SD). One-way ANOVA test was used, followed either by Tukey’s post hoc test to assess statistical significance between four groups or by Bonferonni’s post hoc test to determine statistical significance between two groups, for oxidative stress parameters, AMPK, and adiponectin levels. To analyze the data related to the contraction and relaxation processes, two-way ANOVA was used, followed by the Bonferonni post-test. A *p* value of less than 0.05 was considered statistically significant (^#^ DM vs. C, *p* < 0.05; * DM vs. DM + RA/Ro, *p* < 0.05; ^α^ DM + RA vs. DM + Ro, *p* < 0.05) [24,28,37].

## 3. Results

### 3.1. LC–MS Analysis

The results of the LC–MS analysis (Table 2) highlighted important amounts of polyphenols in the composition of the tested the *R. officinalis* extract. Among the identified compounds, there are representatives with well-known and studied biological activities, including both in the class of phenolic acids and flavonoids.

The metabolite identified in the largest amounts in leaves of *R. officinalis* was carnosic acid (63.87 ± 0.53 mg/mL), a diterpenic acid, previously identified in the composition of the species [12,25,28]. Along with it, important amounts of phenolic acids such as rosmarinic (6.68 ± 0.08 mg/mL), caffeic, and ellagic acids were determined, together with flavonoids such as apigenin, luteolin-7-O-glucoside or myricetin, as well as carnosol, among phenolic diterpenes, which were found in important quantities. The vast majority of metabolites were previously identified in the composition of the species [12,38,39], with the exception of flavonoids such as naringenin, myricetin, and chrysin, for which the present study represents the first report.

### 3.2. The Aorta Ring Reactivity

**Figure 1:**
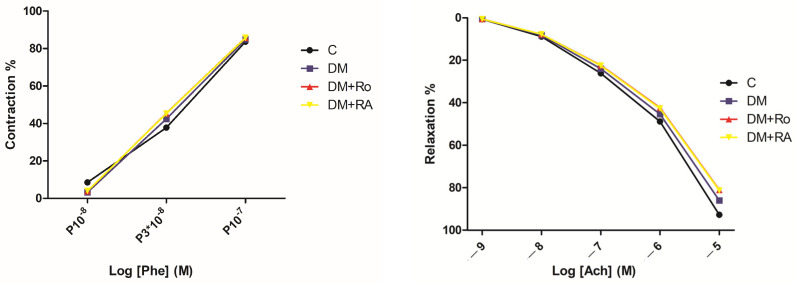
Contractile responses of aortic rings to cumulative concentrations of phenylephrine (Phe, **left**) and relaxation responses of aortic rings to cumulative concentrations of acetylcholine (Ach, **right**). The contractile and relaxing responses were tested for the control group and groups with DM treated with the vehicle, *R. officinalis* extract (Ro), and RA. No statistically significant differences between groups were noticed.

The aorta rings’ reactivity to phenylephrine and acetylcholine was assessed in order to evaluate the vasoconstrictor response mediated by phenylephrine and the endothelium-dependent relaxation induced by acetylcholine (Ach). The aortic fragments were maintained at a resting tension of 1.5–2 g for one hour by changing the KHS solution every 20 min. After confirming endothelial integrity, progressively increasing concentrations, phenylephrine and subsequently acetylcholine, both in concentrations from 10^−9^ to 3 × 10^−5^ M, were added. In this way, maximal endothelium-dependent (acetylcholine-mediated) relaxation of the thoracic aorta precontracted with phenylephrine was determined [40]. In order to evaluate the effect of RA and the *R. officinalis* extract on vascular reactivity, the rings harvested from the two groups were incubated for 60 min with KHS. The results were compared with aortic rings taken from the DM group and from the control group, without diabetes. The results obtained showed that the *R. officinalis* extract and RA tended to improve the relaxation response to Ach 10^−9^–10^−5^ M but without the differences with aortas from DM and control groups (Figure 1). These data suggested low amounts of NO, a vascular relaxant, after the addition of this dose of acetylcholine. Moreover, a short duration of treatment with vasoprotective agents or low doses can influence the contractile activity of the vessels. Possibly, oxidative stress changes are incipient; they trigger the mobilization of antioxidant defense and the regulation of redox imbalance, which may prevent the impairment of the vasoconstrictor or vasorelaxant functions of the vascular wall.

### 3.3. Biochemical Assessment

Regarding the effects of the *R. officinalis* extract and RA on the aorta and heart oxidative stress, the malondialdehyde, GSH and GSSH levels, and superoxide dismutase activity were quantified.

**Figure 2:**
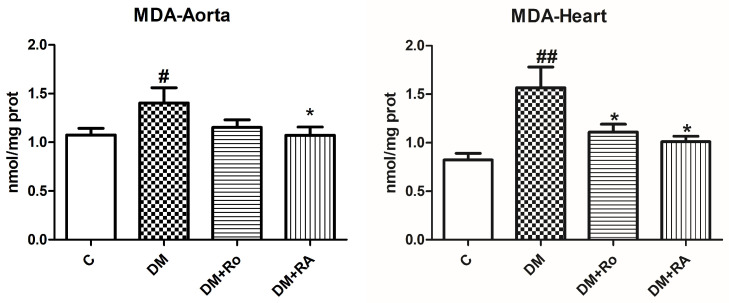
Malondialdehyde (MDA) levels in the aorta and heart of diabetic rats. MDA levels increased after STZ administration (# *p* < 0.05, ## *p* < 0.05) and diminished after *R. officinalis* extract and RA (* *p* < 0.05) administration. Each group consisted of 9 rats. Results are expressed as mean ± SD.

Malondialdehyde (MDA) levels increased in the aorta and heart homogenates of animals with DM compared to the control (*p* < 0.05). Lipid peroxidation was significantly decreased both in the aorta after RA administration (DM vs. DM + RA, *p* < 0.05) and heart (DM vs. DM + RA, *p* < 0.05). The same beneficial effect was found after the *R. officinalis* extract administration (DM vs. DM + Ro, *p* < 0.05 (Figure 2).

**Figure 3:**
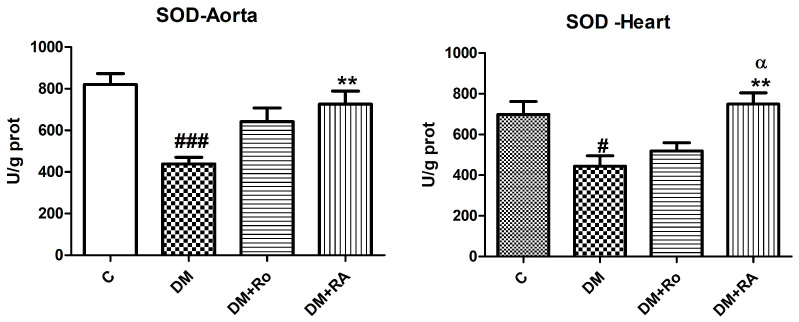
Superoxide dismutase (SOD) activity in the aorta and heart of diabetic rats. SOD activity decreased after STZ administration (### *p* < 0.001 in the aorta and # *p* < 0.05 in the heart) and increased after *R. officinalis* extract (** *p* < 0.05) and RA (DM + RA vs DM + Ro, α, *p* < 0.05) administration. Each group consisted of 9 rats. Results are expressed as mean ± SD.

Superoxide dismutase activity decreased significantly in the aorta homogenates compared to control (*p* < 0.001) and heart tissues (*p* < 0.05) of diabetic animals treated with vehicle. Administration of RA significantly amplified SOD activity in the heart and aorta of diabetic animals (DM vs. DM + RA, *p* < 0.01) compared to animals with DM treated with a vehicle. RA, compared to the *R. officinalis* extract, had beneficial effects on the heart and aorta of diabetic rats, by increasing SOD activities (DM + Ro vs. DM + RA, *p* < 0.05) (Figure 3).

**Figure 4:**
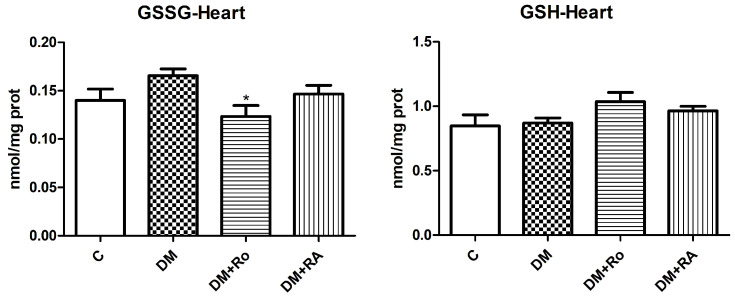
The level of oxidized glutathione (GSSG) in the heart and aorta homogenates of animals with experimentally induced DM. GSSG levels increased after STZ administration but insignificantly reduced after *R. officinalis* extract (* *p* < 0.05) administration. Each group consisted of 9 rats. Results are expressed as mean ± SD.

The treatment with *R. officinalis* significantly reduced the oxidized glutathione (GSSG) levels in the heart of diabetic animals (DM vs. DM + Ro, *p* < 0.05) compared to the DM group (Figure 4).

**Figure 5:**
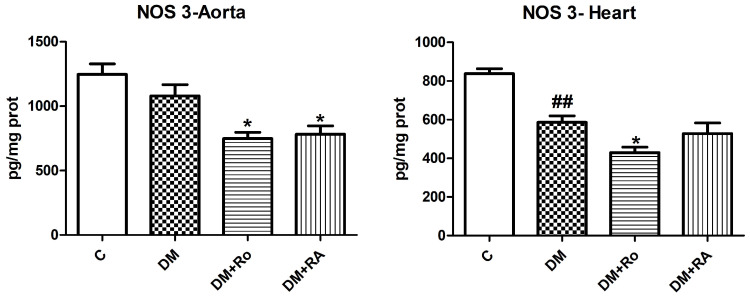
Nitric oxide synthase 3 (NOS 3) levels in the aorta and heart of animals with experimentally induced DM. NOS3 levels decreased after STZ administration (## *p* < 0.01) and further decreased after *R. officinalis* extract administration (* *p* < 0.05) and RA (*p* < 0.05) administration. Each group consisted of 9 rats. Results are expressed as mean ± SD.

The nitric oxide synthetase 3 (NOS 3) levels diminished in the heart homogenates of animals with DM (*p* < 0.001 vs. control). NOS3 levels also decreased significantly in the aorta of animals with DM treated with *R. officinalis*/RA compared to animals with DM (DM vs. DM + Ro, DM vs. DM + RA, *p* < 0.01) and in the heart of animals with DM treated with the *R. officinalis* extract compared to animals with DM (DM vs. DM + Ro; *p* < 0.01) (Figure 5).

**Figure 6:**
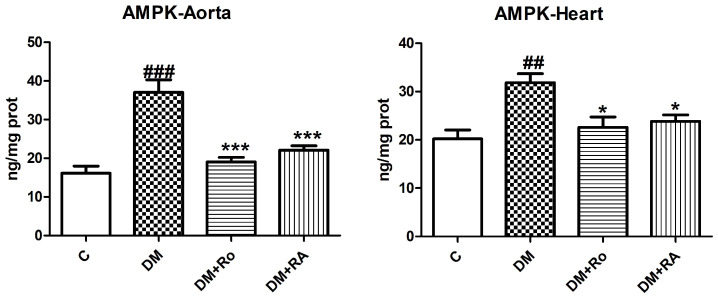
AMPK levels in aorta and heart homogenates of animals with experimentally induced DM. Levels of activated AMP protein kinase (AMPK) in the aorta and heart of diabetic rats, increased after STZ administration (##, ### *p* < 0.05) and diminished after *R. officinalis* extract (*, *** *p* < 0.001) and RA (*, *** *p* < 0.05) administration. Each group consisted of 9 rats. Results are expressed as mean ± SD.

Activated AMP protein kinase (AMPK) secretion increased significantly after streptozotocin administration (DM vs. C, *p* < 0.05), while the administration of the *R. officinalis* extract and RA reduced AMPK levels in aorta and heart homogenates (DM vs. DM + Ro, *p* < 0.001; DM vs. DM + RA, *p* < 0.05) (Figure 6).

**Figure 7:**
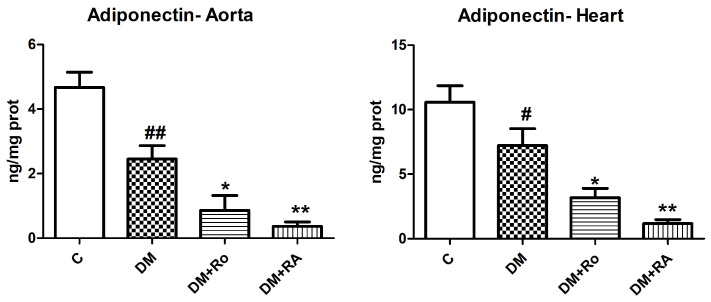
Adiponectin levels in heart and aorta homogenates in DM and rats with DM treated with *R. officinalis* and RA. Adiponectin levels decreased in the aorta and heart homogenates of DM rats (#, ## *p* < 0.01) and after the administration of the *R. officinalis* extract and RA (*, ** *p* < 0.05). Each group consisted of 9 rats. Results are expressed as mean ± SD.

Adiponectin decreased significantly in the aorta and heart of diabetic animals compared to the control group (*p* < 0.01), as well as after the administration of the *R. officinalis* extract and RA (*p* < 0.05) (Figure 7).

### 3.4. Transcription Factors Evaluation

**Figure 8:**
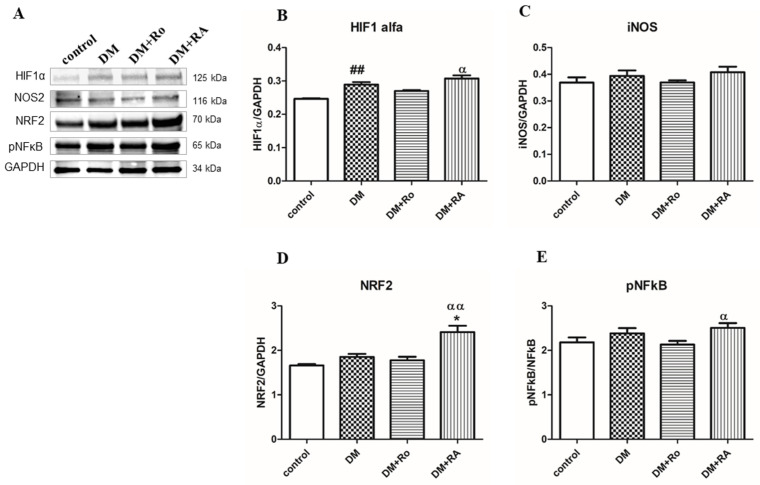
The effects of the *R. officinalis* extract and RA administration on the expression of HIF 1α, iNOS, NRF2, and pNF-κB in the heart. (**A**) Expression of HIF 1α, iNOS, NRF2, and pNF-κB was analyzed using Western blotting (WB). (**B**) HIF 1α, (**C**) iNOS, (**D**) NRF2, and (**E**) pNF-κB. Image analysis of Western blot bands was carried out using densitometry, and the results were normalized to GAPDH. * DM vs. DM + Ro/DM + RA; ^α^ DM + Ro vs. DM + RA. Results are expressed as mean (n = 3) ± SD; *^, α^ *p* < 0.05; ^##, αα^ *p* < 0.01.

The effects of the *R. officinalis* extract and RA on the expression of HIF 1α, iNOS, NRF2, and pNF-κB in the heart homogenates are illustrated in Figure 8. DM significantly upregulated the expressions of the HIF 1α (DM vs. control, *p* < 0.01) (B) and tended to increase the expression of iNOS, NRF2, and pNFkB; however, the differences were not statistically significant (DM vs. control, *p* > 0.05) (C, D, E). RA significantly stimulated the levels of HIF 1α (DM + RA vs. DM + Ro, *p* < 0.05; (B)), NRF2 (DM + RA vs. DM + Ro, *p* < 0.01; (D); DM + RA vs. DM, *p* < 0.05; (D)), and pNFkB (DM + RA vs. DM + Ro, *p* < 0.05; (C)) in the heart. RA increased the expression of NRF2 in the cord homogenates of animals with DM compared to animals with DM without treatment (*p* < 0.05) and induced HIF1 alfa and activation of NFkB compared to animals with DM and treated with Ro (*p* < 0.01 and *p* < 0.05) (Figure 8).

**Figure 9:**
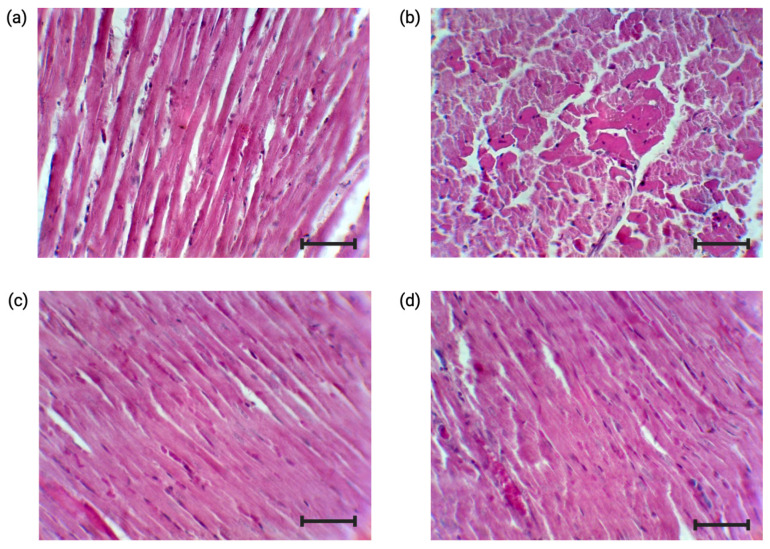
Representative photomicrographs of the hearts of the four experimental groups. Scale bar = 25 µm, 400× magnification, staining with HE. (**a**) Control, (**b**) DM, (**c**) DM + *R. officinalis* extract, (**d**) DM + RA.

The diabetic status induced the formation of eosinophilic bodies and a slight disorganization of the cardiomyocytes (Figure 9b). The administration of the extract (Figure 9c) and RA (Figure 9d) on a diabetic background maintained the histological appearance of the myocardium similar to that of the control (Figure 9a).

## 4. Discussion

Oxidative stress is represented by the imbalance between the production of reactive oxygen species (ROS) and the antioxidant defense, being associated with changes in the normal production of cardioprotective and vasoprotective molecules and damage to the heart and vessels. In our study, DM is accompanied by a pro-oxidative status demonstrated by an increased level of MDA and GSSG and a reduced activity of SOD, both in aortic and cardiac tissue homogenates and high expression of HIF 1α in the heart homogenates. The administration of the extract and RA improved the oxidative status, with a reduction in MDA generation, especially in the cardiac tissue. Additionally, the *R. officinalis* extract diminished the level of GSSG in the heart, while RA reduced MDA formation in the aorta homogenate. The improvement of SOD activity in the presence of RA is related to increased expression of nuclear erythroid 2-related factor 2 (NRF2). This transcription factor belongs to the basic leucine zipper and cap “n” collar family (CNC-bZip protein family) and is known as a complex regulator of metabolic reactions (such as lipid synthesis and catabolism, carbohydrate metabolism and the pentose phosphate pathway, iron metabolism, and storage), of protein homeostasis (the synthesis, folding, stability and autophagy of proteins), of cellular redox homeostasis (GSH synthesis, peroxide reduction, xenobiotic/drug-metabolizing enzymes and membrane transporters), and of inflammation and immunity [41,42,43]. It is noteworthy that NRF2 is regarded as an oxidative stress-activated transcription factor, thus regulating the basal and stress-inducible expression of genes encoding glutathione-based and thioredoxin-based antioxidant system [44]. Natural phytochemicals, such as sulforaphane, resveratrol, curcumin, luteolin, carnosic acid, or apigenin, increase NRF2 activity and induce the target genes [45]. Accordingly, Dodson et al. mentioned that the pharmacological induction of NRF2 with cinnamaldehyde (the primary component of cinnamon bark oil), sulforaphane (found in cruciferous vegetables), and naringenin (a flavanone from the flavonoid group of polyphenols), and significantly diminished the ROS production and increased SOD, CAT, GPx, G6pd, and GST activities. Therefore, indeed, we reached similar conclusions to the literature, regarding the NRF2 activation, and, therefore, the antioxidant effect of the *R. officinalis* extract and RA, in a DM-animal model [41]. In our study, NRF2 was highly expressed in heart tissue after RA administration in parallel with the improvement of SOD activity, suggesting that RA is involved in the upregulation of redox homeostasis.

As reported previously, the mechanism underlying the antioxidant effects of the *R. officinalis* extract has been attributed to its chemical metabolites, such as carnosol, carnosic, rosmarinic, and caffeic acid [46,47].

The level of NOS3 decreased in the heart of diabetic rats, an effect highlighted by *R. officinalis*. The same evolutionary pattern was observed in the aorta homogenates in groups treated with *R. officinalis* and RA. It is known that nitric oxide (NO) produced by NOS has an essential role in maintaining the homeostasis of blood vessels. NO is secreted by endothelium from L-arginine under the action of nitric oxide synthase (NOS) and diffuses into cardiac muscle cells, where it activates guanylate cyclase and generates cGMP with a muscle-relaxing effect [48,49]. Generally, NOS2 is an inducible enzyme (iNOS) involved in NO production after inflammatory stimuli or a prooxidant environment while endothelial NOS (eNOS or NOS3) is implicated in the basal secretion of NO and its physiological effects. Inducible nitric oxide synthase, whose expression is stimulated by proinflammatory cytokines, obesity, free fatty acids, hyperglycemia, endotoxins, or oxidative stress, can be identified in the pathologic conditions in various tissues such as skeletal muscle, adipose tissue, or liver [50,51], while NOS3 is identified in physiological conditions.

As is widely known, NF-κB is considered a time- and concentration-dependent-regulator of iNOS or NOS2 expression, induced by LPS, IL-1β, or TNFα in cardiovascular tissues [50,52,53]. There is also scientific evidence that HIF-1 is essential for iNOS expression in myocardial cells [54,55]. Therefore, in the current study, we evaluated the effect of the *R. officinalis* extract and RA on iNOS (NOS2), pNFkB, and HIF1 α expressions in a DM-animal model. The results showed increased expression of HIF1 α in the DM group, an effect that is amplified by RA. The same effect of RA was found on pNFkB expression. It is considered that iNOS-mediated peroxynitrite stimulates the high glucose-induced inflammatory response in diabetic vascular tissues, as iNOS expression is markedly induced by inflammation and oxidative stress [56]. Endothelial nitric oxide synthase (eNOS) is responsible in the vascular wall for the basal synthesis of NO, which is involved in vascular tone regulation, platelet aggregation, cellular proliferation, and cytoprotection in microvasculature [57]. The alteration of NOS3 activity is accompanied by the reduction in NO bioavailability and endothelial dysfunction, further increasing the cardiovascular risk. However, in many cases, NO generation is associated with excessive superoxide anion production, especially in atherosclerosis or inflammation, and, consequently, with the formation of peroxynitrite, an extremely cytotoxic compound [58,59]. It was demonstrated that NOS3 exerts dual activity in DM, and produces high levels of NO in parallel with the generation of large amounts of superoxide anion and peroxynitrite. Therefore, in some pathologic conditions, therapies that reduce NOS 3 overexpression improve nitrosative and oxidative stress and reduce damage to arteries [60,61] like in our experiment.

Moreover, adiponectin is considered to be involved in stimulating the production of NO in endothelial cells [62]. The activation of AMPK in endothelial cells stimulates NO production due to phosphorylation and activation of NOS3 [63]. AMPK is an important enzyme in DM because it improves insulin sensitivity, promotes the survival of islet β cells, and reduces their apoptosis, inflammation, and oxidative stress [64]. In addition, it improves the uptake and metabolism of glucose in the cell, thus reducing insulin resistance and DM complications. AMPK is activated by various stressors when ATP in the cell decreases. It appears that AMPK1 activation induces aortic vasorelaxation in relation to NO production by NOS3. In our study, in DM, the enzyme levels increase adaptively, an effect diminished by the tested extract and by the RA, both in the heart homogenate and in the aortic tissue. These results suggest that the administered *R. officinalis* extract or RA did not improve the activity of this enzyme and basal secretion of NO in heart and vascular tissue. It has been reported that natural products, such as resveratrol, berberine, ginsenosides, quercetin, curcumin, or naringenin may exert antioxidative, anti-inflammatory, cardioprotective, and endothelial protective effects through the activation of various AMPK signaling pathways (e.g., AMPK/Nrf2/HO-1, AMPK-SIRT3) [65]. Additionally, Vlavcheski et al. mentioned the AMPK activation, both regarding *R. officinalis* extract and rosmarinic acid [66]. Conversely, with regard to the administration of the extract and RA, our results were contradictory with the above-mentioned data probably due to the dose used, the short duration of the experiment, or the involvement of other intricate mechanisms.

In parallel, adiponectin is reduced both in the aorta and in the cardiac tissue homogenate in DM and decreases even more in the case of *R. officinalis* extract and RA administration. Adiponectin is a cardioprotective adipokine downregulated in type 2 DM (T2DM) and, therefore, its downregulation indicates intrinsic cardioprotective properties [67]. The effect on the level of this cytokine limits the effectiveness of tested compounds in DM, with one explanation being the too-small dose and the short duration of the administration of the extract or RA.

Our results indicate that the administration of the *R. officinalis* extract and A alleviated oxidative stress, by decreasing the level of MDA and GSSG and increasing the level of SOD, proving that vascular and cardiac antioxidant properties without affecting intrinsic mechanisms are involved in reducing blood glucose in DM or in vascular protection.

Although there is scientific evidence that diabetic hyperglycemia may decrease the HIF-1α expression, the activation of HIF-1 signaling by a carbohydrate response element-binding protein has been observed in glomerular mesangial cells, thus suggesting a cell-type regulatory pattern of HIF-1 in diabetes mellitus [68,69,70].

RA inhibited the expression levels of NF-κB in STZ-treated rats, as mentioned by some authors [71]. Similar studies have proved that *R. officinalis* methanolic extract increased total antioxidant capacity and the expression of superoxide dismutase (SOD), with the subsequent decrease in malondialdehyde levels improving nitric oxide metabolism in rats with myocardial infarction. These biological activities were linked to their polyphenolic compounds [72]. Carnosic acid, the main compound found in the composition of the tested extract, together with carnosol, its derivative, is strongly related to the antioxidant activity [6,7,11,14]. Moreover, the antioxidant activity of phenolic acids (rosmarinic, caffeic, and ellagic acids) in the composition of the tested extract [3,5,6,21].

RA proved to protect aortic endothelial function and structure against diabetes-induced damage, directly related to their antioxidant and anti-inflammatory effects [73]. Moreover, it attenuated cardiac fibrosis following long-term pressure overload by the AMPKα/Smad3 signaling pathway [74]. These studies offered premises for our study, showing the potential of the *R. officinalis* extract and RA in the protection of cardiac tissue probably in other doses administered over a longer period of time. In this way, the present study finds its novelty, namely, that to the best of our knowledge, it is the first study comparing the effects of the extract and its main phenolic acid in the protection against the oxidative stress induced by DM in rats.

## 5. Conclusions

The results of the present study showed that the administration of the *R. officinalis* extract and RA alleviated oxidative stress by decreasing the level of MDA and GSSG and increasing SOD activity, proving the existence of antioxidant properties in the heart or aorta in DM. In parallel, RA induced the synthesis of a transcription factor involved in the upregulation of genes encoding the glutathione-based and thioredoxin-based antioxidant system. The *R. officinalis* extract and RA did not improve intrinsic mechanisms involved in heart and vascular protection, especially in NO production or in the regulation of glucose and fatty acid metabolism. RA and the *R. officinalis* extract exert mainly antioxidant effects, improving the expression of NRF2 and HIF1 but negatively influencing the AMPK and adiponectin levels in this experimental model and in these doses. However, the results provide evidence for the use of the *R. officinalis* extract, as well as RA as adjuvants in the treatment of DM due to their antioxidant activity.

## Figures and Tables

**Table 1:** Gradient concentration of the LC–MS mobile phase.

Time (min)	% Methanol	% Water	% of 2% Formic Acid in Water
0.00	5	90	5
3.00	15	70	15
6.00	15	70	15
9.00	21	58	21
13.00	21	58	21
18.00	30	41	29
22.00	30	41	29
26.00	50	0	50
29.00	50	0	50
29.01	5	90	5
35.00	5	90	5

**Table 2:** Identification and quantification of polyphenolic metabolites in the composition of the *Rosmarinus officinalis* extract.

Compound	Retention Time (min)		*m*/*z* and Main Transitions		Concentration (mg/mL)
	Standard	Separated	Standard	Separated	*R. officinalis*
Caffeic acid	13.8	13.7	179.0 > 135.0	179.0 > 135.0	5.25 ± 0.06
Carnosic acid	32.0	32.1	331.2 > 285.1	331.2 > 285.1	63.87 ± 0.53
Chlorogenic acid	12.0	12.0	353.0 > 191.0	353.0 > 191.0	1.29 ± 0.2
*trans-p*-coumaric acid	17.5	17.5	163.0 > 119.0	163.0 > 119.0	0.66 ± 0.01
Ellagic acid	27.3	27.3	301.0 > 185.0	301.0 > 185.0	12.74 ± 0.12
Gallic acid	7.0	7.0	168.9 > 125.0	168.9 > 125.0	0.04 ± 0.01
Rosmarinic acid	21.4	21.6	358.9 > 161.0	358.9 > 161.0	6.68 ± 0.08
Ferulic acid	18.4	18.5	193.0 > 134.0	193.0 > 134.0	0.06 ± 0.01
Salicylic acid	23.5	23.5	137.0 > 93.0	137.0 > 93.0	-
Apigenin	28.2	28.1	269.0 > 117.0	269.0 > 117.0	3.42 ± 0.02
Carnosol	30.7	30.4	329.1 > 285.1	329.1 > 285.1	3.06 ± 0.03
Chrysin	29.7	29.7	253.0 > 143.0	253.0 > 143.0	-
Hesperetin	27.1	27.0	301.0 > 164.0	301.0 > 164.0	0.44 ± 0.06
Hyperoside	20.3	20.4	463.1 > 300.0	463.1 > 300.0	0.81 ± 0.09
Kaempferol	27.9	27.9	285.0 > 187.0	285.0 > 187.0	0.12 ± 0.02
Luteolin-7-*O*-glucoside	19.9	19.9	447.0 > 284.9	447.0 > 284.9	1.36 ± 0.02
Luteolin	26.9	26.8	287.0 > 153.0	287.0 > 153.0	0.91 ± 0.01
Myricetin	13.6	13.6	317.0 > 179.0	317.0 > 179.0	5.01 ± 0.04
Naringenin	26.3	26.3	271.0 > 119.0	271.0 > 119.0	0.03 ± 0.01
Quercetin	25.4	25.5	300.9 > 151.0	300.9 > 151.0	0.04 ± 0.01
Rutoside	20.3	20.3	609.0 > 300.0	609.0 > 300.0	0.12 ± 0.02
Vitexin	18.4	18.5	431.0 > 311.0	431.0 > 311.0	-

Note: LOQ = limit of quantification. The results represent the mean of 3 determinations ± the standard deviation (n = 3).

## Data Availability

The original contributions presented in the study are included in the article, further inquiries can be directed to the corresponding author/s.
